# The modified lateral window technique for retrieving root stumps from the maxillary sinus: Case report

**DOI:** 10.1097/MD.0000000000041288

**Published:** 2025-02-07

**Authors:** Chongyuan Liu, Lidi Cheng, Qingying Xu, Fen Yang, Fengjia Liu

**Affiliations:** a Department of Stomatology, Huzhou Central Hospital, Fifth School of Clinical Medicine of Zhejiang Chinese Medical University, Huzhou, Zhejiang, China; b Department of Stomatology, Huzhou Central Hospital, Affiliated Central Hospital of Huzhou University, Huzhou, Zhejiang, China; c Department of Stomatology, Changxing County Traditional Chinese Medicine Hospital, Huzhou, Zhejiang, China; d Department of Stomatology, Nanxun Traditional Chinese Medicine Hospital, Huzhou, Zhejiang, China.

**Keywords:** intranasal endoscopic approach, lateral window approach, maxillary sinus, root stump, 3-dimensional reconstruction, tooth extraction

## Abstract

**Rationale:**

Tooth extraction is a common surgical procedure in oral and maxillofacial surgery. Extraction of the upper molar teeth may result in root fracture for anatomical reasons, leading to the migration of fractured roots into the maxillary sinus. This can cause serious complications such as oroantral fistula, sinusitis, cellulitis, and subdural abscesses. To mitigate the risk of these complications, it is important to promptly remove the root stumps from the maxillary sinus and preserve alveolar bone for subsequent prosthetic rehabilitation.

**Patient concerns:**

A 30-year-old male patient presented for the removal of a root stump that had migrated into the maxillary sinus following the extraction of a right upper posterior tooth. A 28-year-old male patient sought dental implant restoration following the extraction of an upper left posterior tooth. Imaging revealed a residual root in the maxillary sinus, which could affect subsequent restoration.

**Diagnoses:**

Residual root in the maxillary sinus.

**Interventions:**

Two cases of root stumps displaced into the maxillary sinus were treated using the modified lateral window technique, with good recovery. Besides, the second patient underwent a successful implant placement 4 months postoperatively.

**Outcomes:**

Two cases of root stumps displaced into the maxillary sinus were removed though the modified lateral window technique, with good recovery. The second patient, who underwent implant restoration 4 months postoperatively, demonstrated good implant stability during subsequent follow-up assessments.

**Lessons:**

This paper presents a modified lateral window technique, which is used to remove the stump in the maxillary sinus, resulting in a good postoperative recovery and providing sufficient bone for implant placement. It should be noted that this surgical approach is not applicable to the scenario “the root is free within the sinus”, precise localization of the foreign body becomes challenging, and alternative surgical methods may need to be considered.

## 1. Introduction

Tooth extraction is one of the most common oral and maxillofacial surgeries. When extracting upper molar teeth, due to specific anatomical reasons, root fracture often occurs, and the fractured roots can easily migrate into the maxillary sinus. Furthermore, this can lead to severe complications such as oroantral fistula, sinusitis, cellulitis, and subdural abscess.^[1-3]^ To prevent these conditions, it is crucial to promptly remove the root stumps from the maxillary sinus and preserve as much alveolar bone as possible for future implant placements. According to current literature, there are 4 surgical approaches to retrieve root stumps from the maxillary sinus. First, the alveolar crest approach, which may damage the maxillary sinus floor and increase the risk of oroantral fistula. Second, the Caldwell–Luc approach, which carries a risk of damaging the infraorbital nerve, potentially leading to facial numbness and sensory abnormalities.^[4]^ Third, the intranasal endoscopic approach, which requires a specialist with experience in endoscopic operations.^[5,6]^ Fourth, the lateral sinus window approach with the use of a resorbable membrane, which provides good surgical visibility and does not affect subsequent restorations.^[7-9]^

This study proposes a modified lateral window technique for the extraction of root stumps from the maxillary sinus, aiming to preserve as much bone at the sinus floor as possible for future implants, while also reducing surgical risks, simplifying the procedure, and shortening the operation time. In this report, 2 cases of root stumps displaced into the maxillary sinus were treated using the modified lateral window technique, with good recovery. The second patient underwent a successful implant placement 4 months postoperatively, demonstrating good implant stability. Furthermore, we discuss different techniques for removing displaced root stumps from the maxillary sinus and provide a brief review of the literature.

## 2. Case report

### 2.1. Case 1

**Figure 1.**
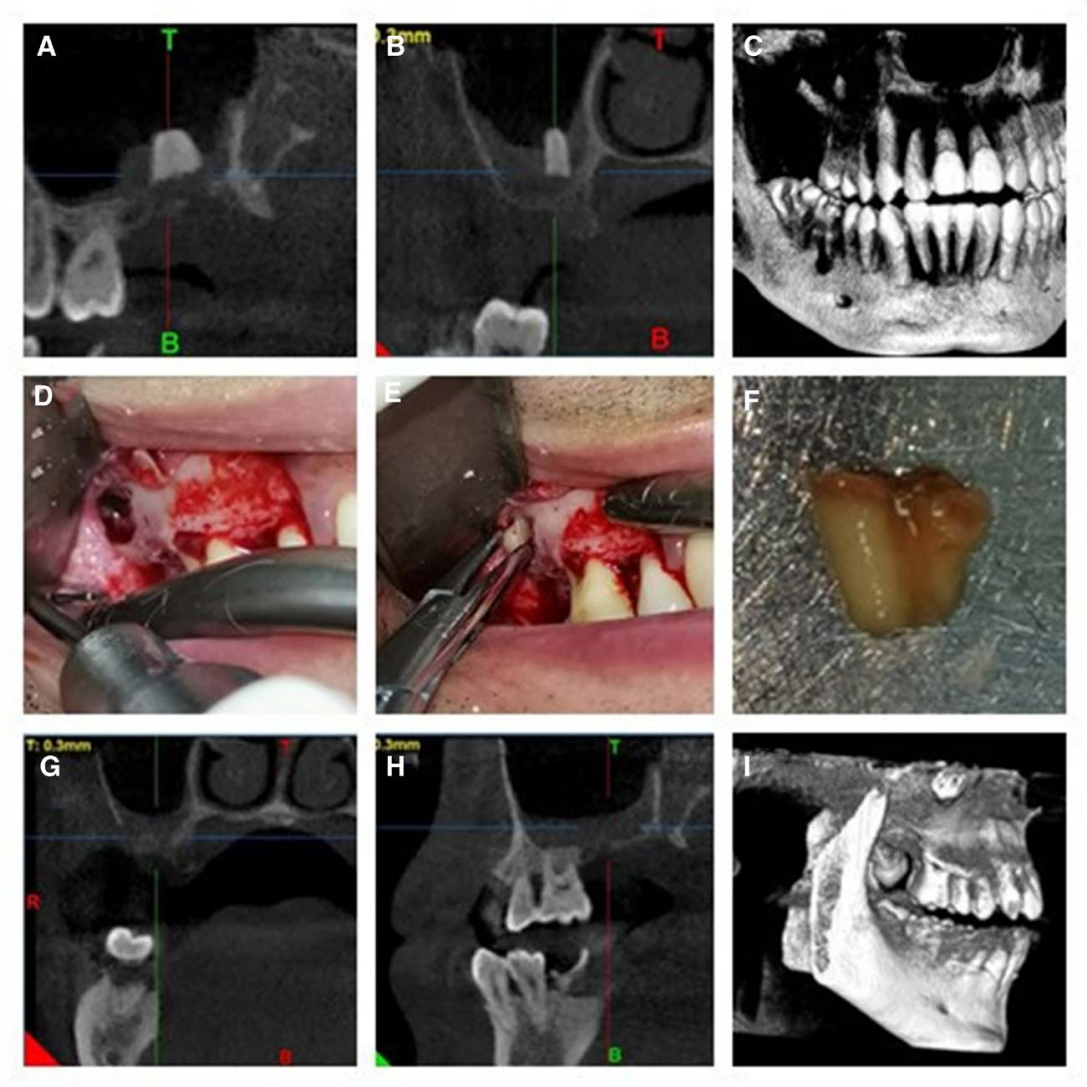
CBCT images, intraoral photographs, and the extracted root stump of case 1. (A and B) CBCT images showing a root stump within the right maxillary sinus. (C) Three-dimensional reconstruction illustrating a root stump in the right maxillary sinus. (D) Ultrasonic bone scalpel creates a window in the lateral wall of the right maxillary sinus and elevation of the sinus membrane. (E) Removal of the root stump using hemostatic forceps. (F) The extracted root stump. (G and H) Postoperative CBCT images showing no foreign bodies in the right maxillary sinus with minor effusion. (I) Three-dimensional reconstruction displaying the bone window in the lateral wall of the right maxillary sinus. CBCT = cone-beam computed tomography.

A 30-year-old male was referred to our department of oral and maxillofacial surgery for the removal of a root stump that had migrated into the maxillary sinus following an extraction of the right upper posterior tooth performed 2 months prior at another hospital. Despite undergoing anti-inflammatory treatment, the root stump remained within the sinus. Preoperative cone-beam computed tomography (CBCT) scans in both upright and supine positions revealed the presence of the root stump, which was adherent to the thickened mucosa of the right maxillary sinus (Fig. 1A and B). The 3-dimensional reconstruction located the root at the bottom of the right maxillary sinus, near the palatal bone wall (Fig. 1C).

After informing the patient of the procedure and associated risks, and obtaining his informed consent, comprehensive preoperative assessments were conducted to rule out any contraindications to surgery. The modified lateral window technique was then employed to remove the root stump. One hour before the procedure, the patient was administered 1.2 g of amoxicillin-clavulanate potassium intravenously. Under local anesthesia with articaine and 1:100,000 epinephrine, an L-shaped incision was made from the area of the premolars to the maxillary tuberosity, and the flap was reflected to expose the lateral wall of the right maxillary sinus. At a level estimated to be parallel to the root stump and 2 mm above the floor of the sinus, a window was created using an ultrasonic bone scalpel (Setlec, Piezotome Solo, France) closely matching the size of the root stump. The sinus membrane was gently elevated using a sinus lift elevator, taking care not to perforate it. Once the periosteal stripping reached below the root stump, it was palpable, and the membrane was punctured at this location to move the root under the membrane and slowly toward the bone window, where it was removed with hemostatic forceps (Fig. 1D–F). The surgical site was sutured and postoperative imaging confirmed that no root stumps were left behind. (Fig. 1G–I) The entire procedure took ≈20 minutes.

Postoperatively, the patient received intravenous amoxicillin-clavulanate potassium 1.2 g twice daily for 5 days and was advised to rinse with chlorhexidine for 2 weeks. The patient was instructed not to blow his nose for 4 weeks. Follow-up indicated good healing of the surgical site with no complications.

### 2.2. Case 2

**Figure 2.**
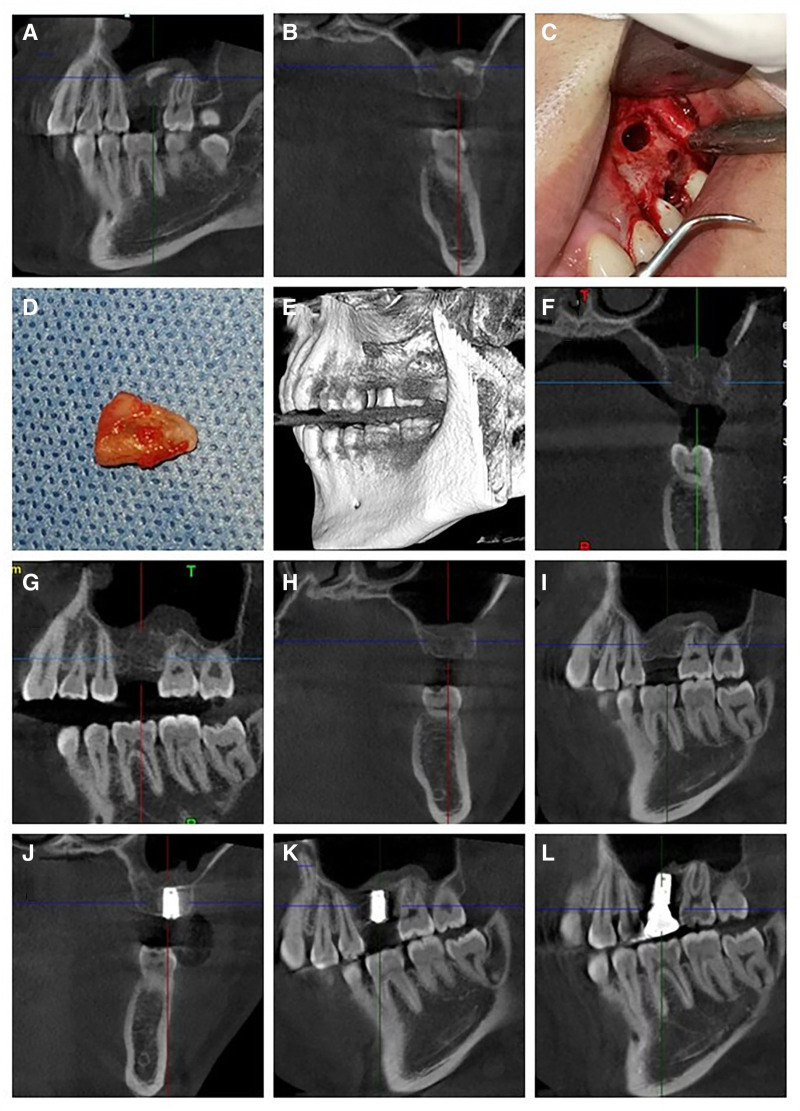
CBCT images, intraoral photographs, and the extracted root stump of case 2. (A and B) CBCT images showing the root stump within the left maxillary sinus. (C) Elevation and separation of the maxillary sinus membrane following lateral window opening on the left maxillary sinus. (D) The extracted root stump. (E) Three-dimensional reconstruction showing the bone window on the lateral wall of the left maxillary sinus. (F and G) Postoperative CBCT images showing no foreign objects within the left maxillary sinus, with no significant effusion. (H and I) Good healing of the extraction site at position 26, 4 months postoperatively. (J and K) Routine implant placement at site 26, 4 months postoperatively. (L) Completion of implant restoration at site 26. CBCT = cone-beam computed tomography.

A 28-year-old male patient sought dental implant restoration for an upper left posterior tooth extraction. Preimplant radiographs revealed a residual root in the left maxillary sinus, prompting a visit to our oral and maxillofacial surgery department. Oral CBCT imaging showed the residual root located between the sinus membrane and the bony floor of the maxillary sinus, near the cheek side wall on the sagittal view, without penetrating the mucosa (Fig. 2A and B). After excluding any preoperative contraindications and obtaining informed consent, the patient underwent a modified lateral window sinus lift procedure.

One hour before the surgery, the patient was administered 1.2 g of amoxicillin-clavulanate potassium intravenously. The procedure was carried out under local anesthesia with articaine and 1:100,000 epinephrine. An “L” shaped incision was made from the area of the premolars to the maxillary tuberosity. Using an ultrasonic bone scalpel, a window was created 2 mm above the floor of the sinus, and the sinus membrane was elevated with an elevator to reach the root stump. A curette was used to lift the root stump under the membrane and slowly move it toward the bone window, where it was removed with hemostatic forceps (Fig. 2C and D). The postoperative CBCT images confirmed that no root stumps were left behind (Fig. 2E–G). The total operation time was approximately 18 minutes. Due to poor healing of the extraction socket at position 26, implant placement was not performed simultaneously. Postoperatively, the patient was instructed not to blow his nose for 4 weeks to prevent infection. Four months after the removal of the root stump, the socket at position 26 healed well with no complications, (Fig. 2H and I), and a dental implant was placed. Follow-up showed good stability of the implant (Fig. 2J–L).

## 3. Discussion

In clinical practice, the roots of some premolars and molars are located within the maxillary sinus, covered only by a thin bone plate or even just the sinus membrane. Extractions in these patients require careful handling to prevent root fractures. Previous studies have shown that male patients are more likely to experience root migration into the maxillary sinus during upper molar extractions.^[10]^

Currently, there are 4 clinical approaches to retrieve roots from the maxillary sinus. The first is via the alveolar crest approach, which can lead to loss of bone at the floor of the maxillary sinus, necessitating additional clinical procedures to restore bone volume, complicating and lengthening the recovery, and increasing the risk of oroantral fistulas and postoperative infections.^[11]^ The second approach is the Caldwell–Luc approach, which involves using a suction device to extract the root but may damage the infraorbital nerve and requires a clinician experienced in endoscopic operations.^[12]^ The third uses an endoscope through the middle nasal meatus to remove implants from the maxillary sinus. However, recent reports have indicated that clinicians using nasal endoscopy have failed to locate the root within the sinus.^[13]^

**Figure 3.**
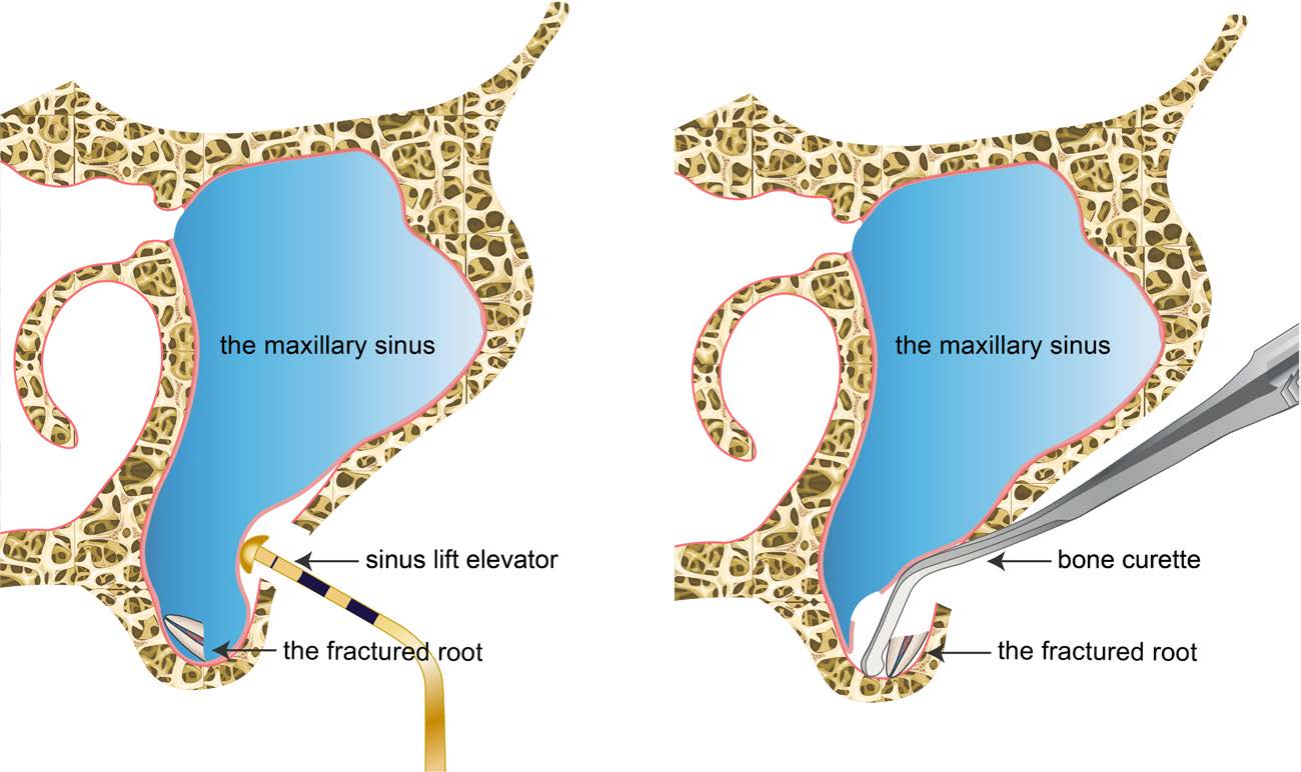
Schematic diagram for the modified lateral window technique.

This study proposes a modified lateral window technique for retrieving roots from the maxillary sinus, constituting the 4th category through a lateral window approach. This technique offers several advantages: First, after preparing the bone window, the sinus membrane is elevated as in conventional sinus lift surgery until the elevator reaches the root, and the membrane is opened at this point to move the root between the elevated membrane and the bony wall of the sinus. The root is then slowly moved toward the bone window and removed with hemostatic forceps, confined within this “tent-like” space and preventing it from becoming “lost” in the sinus (Fig. 3). Second, the lateral window technique is well-mastered by oral implantologists and maxillofacial surgeons without the need for specialized endoscopic training. Third, the lateral window approach allows for direct visualization and extraction of the root under good visual conditions. Fourth, it effectively preserves the bone at the floor of the maxillary sinus, which is beneficial for future implant restorations and avoids the risk of oroantral fistulas.

Roots in the maxillary sinus are categorized into 3 types: First, the root is free within the sinus; second, the root is adherent to the sinus membrane; third, the root is located between the sinus membrane and the alveolar bone.^[6]^ The 2 cases in this study belong to the 2nd and 3rd categories. It should be noted that this technique is suitable for the 2nd and 3rd types of cases. If the root or restoration is freely mobile within the sinus cavity and its position varies with changes in the patient’s posture, precise localization of the foreign body becomes challenging. Consequently, this surgical approach is not applicable to the first scenario “the root is free within the sinus,” and alternative surgical methods may need to be considered if the root is free within the sinus.

## 4. Conclusion

The modified lateral sinus lift technique for retrieving roots from the maxillary sinus shortens surgery time, reduces patient discomfort, requires less specialized surgical equipment and instruments, making it more suitable for dentists without endoscopic experience, and preserves alveolar bone, aiding future implant restorations. Although roots in the maxillary sinus are uncommon, only 2 cases are reported here, the modified lateral sinus lift technique provides an additional surgical option.

## Author contributions

**Conceptualization:** Chongyuan Liu, Lidi Cheng, Fengjia Liu.

**Data curation:** Chongyuan Liu, Lidi Cheng, Qingying Xu, Fen Yang.

**Visualization:** Chongyuan Liu, Lidi Cheng.

**Writing – original draft:** Chongyuan Liu, Lidi Cheng.

**Writing – review & editing:** Qingying Xu, Fen Yang, Fengjia Liu.

**Project administration:** Fengjia Liu.

**Supervision:** Fengjia Liu.
